# Comparison of Risk Stratification by CanAssist Breast Test Performed on Core Needle Biopsies Versus Surgical Specimens in Hormone Receptor-Positive, Her2-Negative Early Breast Cancer

**DOI:** 10.7759/cureus.70054

**Published:** 2024-09-23

**Authors:** Badada Ananthamurthy Savitha, Payal Shrivastava, Rahul Bhagat, Naveen Krishnamoorthy, Deepti K Shivashimpi, Manjiri M Bakre

**Affiliations:** 1 Technical and Analytical Division, OncoStem Diagnostics Pvt. Ltd, Bengaluru, IND; 2 Design and Development, OncoStem Diagnostics Pvt. Ltd, Bengaluru, IND

**Keywords:** canassist breast, concordance between core needle biopsy and surgical specimen, core needle biopsy, early breast cancer, prognostication

## Abstract

Introduction

Core needle biopsies (CNB) are being increasingly utilized for biomarker, prognostic, and predictive testing in breast cancer (BC). CanAssist Breast (CAB) is a prognostic test performed to assess the ‘risk of breast cancer recurrence’ in early-stage hormone receptor-positive, Her2-negative BC patients. CAB segregates tumors as ‘low risk’ or ‘high risk’ for distant recurrence. Risk assessment done by CAB aids in planning and making adjuvant chemotherapy or hormone therapy decisions. CAB is typically performed on surgical specimens (SS). However, performing it on CNB does offer additional insights into tumor biology leading to different strategies for treatment planning; hence, we aimed to compare the risk stratification performance of CAB using CNB versus SS.

Method

We analyzed 103 paired formalin-fixed paraffin-embedded CNB and SS samples from hormone receptor-positive, Her2-negative early BC tissue samples submitted for performing CAB at OncoStem Diagnostics between November 2021 and September 2023. Concordance on ‘risk categories’ of CAB performed on CNB versus SS was reported using overall percentage agreement and Pearson correlation coefficient.

Results

We found excellent overall concordance of 92.2% for CAB risk stratification between paired CNB and SS tumor samples with a strong Pearson correlation coefficient of r= 0.8351 (p< 0.0001) when either SS or CNB was used as the gold standard. In prognostic testing patients with a ‘low risk’ of recurrence may avoid chemotherapy and hence it is crucial to assess the accuracy of CAB in the low-risk category. Additionally, in a real-world scenario, it is more likely that CAB will be performed on CNB first.

Conclusion

CAB when performed on CNB samples showed high concordance with SS thus demonstrating that CNB was a suitable sample for the CanAssist Breast test. The accuracy in the low-risk category is 97.5%, which ensures that physicians can reliably use prognostic information by testing CNB to guide adjuvant therapy decisions.

## Introduction

Breast cancer (BC) is the most prevalent cancer and a major cause of cancer-related deaths globally. BC has ranked as the leading cancer among Indian women with an age-adjusted rate as high as 25.8 per 100, 000 women and a mortality of 13.3 per 100,000 women. There is a trend of increase in incidence with a 39.1 % increase in the age-standardized incidence rate of female breast cancer from 1990 to 2016 [1-3]. Treatment for BC involves a multi-disciplinary approach with surgery, radiation, endocrine therapy, chemotherapy, and now immunotherapy. Choosing optimal adjuvant therapy is crucial in achieving good outcomes and improving survival [1, 4 -5].

Core needle biopsy (CNB) is one of the initial steps in diagnosing invasive BC and treatment planning. Guidelines recommend biological subtyping and hormone receptor testing i.e., estrogen receptor (ER), progesterone receptor (PR), and Her2 on CNB despite concordance rates below 100% between paired CNB and resected SS [4, 6]. It also helps identify patients requiring neoadjuvant chemotherapy (NACT) or neoadjuvant hormonal therapy (NAHT).

CanAssist Breast (CAB) is a validated proteomic-based prognostic test performed on BC samples that predicts the risk of distant recurrence within 5 years of diagnosis for patients with hormone receptor-positive, Her2-negative early BC. CAB uses the immunohistochemistry (IHC) technique to assess the expression of five protein biomarkers (CD44, ABCC4, ABCC11, N-cadherin, and pan-cadherin) on formalin-fixed paraffin-embedded (FFPE) surgically resected tumor tissues. This information in combination with morphometric features (tumor size, histopathological grade, and node status) is incorporated into the proprietary support vector machine learning (SVML)-based algorithm to derive a CAB risk score. CAB segregates patients into two actionable groups using a cut-off of 15.5 to dichotomously segregate patients into a low risk of distant recurrence with a CAB risk score of 15.5 or below; and a high risk of distant recurrence with a CAB risk score above 15.5 [7-9]. CAB has been validated on BC patient cohorts from India, the USA, and Europe demonstrating identical performance on 5-year recurrence risk predictions across multiple races and ethnicities [10-12].

The aim of this study is multi-fold. The primary aim is to analyze the concordance/discordance in risk stratification by CAB when performed on CNB versus SS from hormone receptor-positive, Her2-negative early BC patients. The secondary aim is to establish the sensitivity, specificity, positive predictive values (PPV), and negative predictive value (NPV) of CNB as a sample. Additionally, we seek to identify potential patients who may need retesting with FFPE samples of SS.

## Materials and methods

Patient selection, data collection, and ethics approval

This retrospective data analysis study was performed in line with the principles of the Declaration of Helsinki. Ethics approval was granted by the Sri Venkateshwara Hospital Ethics Committee (ECR/Committee (A/2013/RR-19)). This study is part of CAB testing performed as per the patient’s written consent given in the test requisition form to use, as appropriate, patient data in an anonymized and non-identifiable manner. To protect patient identification - all the patient identifiers were de-identified. Patient’s non-identifiable information including age, gender, clinical parameters, histopathology diagnosis, tumor grade, TNM staging, and hormone receptor status was collected from the test requisition form and patient’s reports submitted for the CAB test.

In this study, the inclusion criteria include the availability of SS and CNB samples in hormone receptor-positive early BC stage (stages I and II) for which information was available about tumor type, age, type of surgery, tumor (size, tumor grade, histopathologic type), and node status. Additionally, the sample submitted should have at least 30% or more invasive tumor content in the FFPE block for adequate assessment of IHC staining on several sections. Also, there should be less than 50% necrosis and hemorrhagic content in the submitted FFPE block/s. Exclusion criteria include hormone receptor-negative tumors, advanced BC (stages III and IV tumors), non-availability of complete information about the type of tumor, age, tumor (size, tumor grade, histopathologic type), and node status. Also, patients with any evidence of local recurrence including chest wall, ipsilateral, or contralateral tumors, or patients treated with neoadjuvant therapy were excluded.

In this study, we analyzed 103 hormone receptor-positive, Her2-negative early BC tissues (stages I and II) for which paired CNB and SS samples that fulfilled the inclusion criteria mentioned earlier. The patient samples were submitted for CAB testing at OncoStem Diagnostics Pvt. Ltd laboratory at Bengaluru between November 2021 and September 2023. All tumor tissue samples were submitted as FFPE blocks. SS includes lumpectomy, breast-conserving surgery (BCS), wide local excision (WLE), mastectomy, and modified radical mastectomy (MRM). Post internal pathological review, the sample was accepted for the CAB test if adequate tumor content was present on the Hematoxylin and Eosin (H&E) review of sections.

Pathology evaluation and CanAssist Breast test

**Figure 1:**
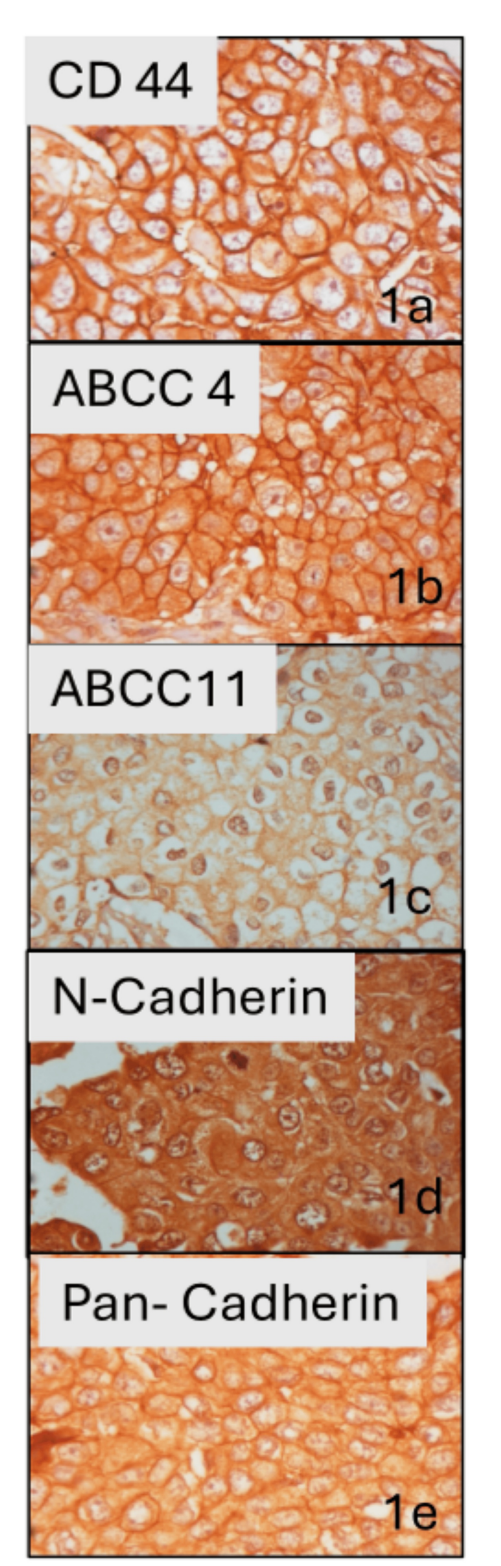
Representative images of IHC staining patterns of CAB biomarkers 1a) CD44, 1b) ABCC4, 1c) ABCC11, 1d) N-cadherin, and 1e) pan-cadherin at 40× magnification. IHC: immunohistochemistry; CAB: CanAssist Breast

Tumor samples from hormone receptor-positive, Her2-negative early BC fixed in 10% neutral buffered formalin and embedded in paraffin were cut into 3-4 µm sections and initially stained with H&E. IHC was performed on consecutive sections for five CAB biomarkers (CD44, ABCC4, ABCC11, N-cadherin, and pan-cadherin) on the automated Ventana platform. IHC grading data by trained oncopathologists, along with node status, tumor size, and tumor grade were used to arrive at the CAB risk score by our proprietary SVML algorithm. Reference images of IHC staining patterns of CD44, ABCC4, ABCC11, N-cadherin, and pan-cadherin are shown in Figure 1. With a 15.5 risk score as a cut-off, patients were categorized to have either ‘low risk’ (0-15.5 risk score) or ‘high risk’ (15.6-100 risk score) for distant cancer recurrence as described earlier [7-8]. CAB was performed on SS first and CNB samples after a two-week washout period in a blinded manner without providing any demographic, tumor staging, or any other clinical information to the oncopathologists.

Statistical analysis

The primary aim of the study was to analyze the concordance of CAB risk categories using CNB and SS. Descriptive statistics were used to summarize the clinicopathological features of the patient cohort. The distribution of the CAB risk score for CNB and SS was mapped with the frequency of patients on the Y-axis and the CAB risk score on the X-axis. Concordance was studied using overall percentage agreement, and correlation was studied using the Pearson correlation coefficient on MedCalc. Statistical graphs and figures were prepared using MedCalc Software version 22.032-64-bit. Pearson’s correlation coefficient values interpreted as 0.00 to 0.10 were considered as negligible correlation, 0.10 to 0.39 as weak correlation, 0.40 to 0.69 as moderate correlation, 0.70 to 0.89 as strong correlation, and 0.90 to 1.00 as very strong correlation [13].

Sensitivity, specificity, NPV, and PPV were also calculated, FFPE tumor blocks from SS were considered the gold standard, and the formulae mentioned below were used [14].

Sensitivity = [True Positive/ (True Positive +False Negative)] X 100,

Specificity = [True Negative/ (False Positive +True Negative)] X 100

PPV = [True Positive / (True Positive +False Positive)] X 100

NPV = [True Negative / (False Negative +True Negative)] X 100

The area under receiver operating characteristic (AUROC) was calculated using MedCalc (binomial exact) [15].

For all CNBs, tumor volume was calculated based on the number of tumor-positive cores multiplied by the average percentage of tumor content in all tumor-positive cores. Based on the tumor volumes derived, the CNB samples were bifurcated into two groups as follows: (1) tumor volume <2 units and (2) tumor volume >2 units. Cohen’s kappa (κ) was used to measure the inter-rater agreement between CNB and SS within the two tumor volume groups. κ values < 0.20 were interpreted as poor agreement, 0.21-0.40 as fair agreement, 0.41-0.60 as moderate agreement, 0.61-0.80 as good agreement, 0.81-0.99 as very good agreement, and 1 as perfect agreement [16].

## Results

The study cohort had 103 patients, with ages ranging from 32 to 76 years and a median age of 58 years. The analysis was performed on all patients with reportable results from the CAB test. Twenty-five (24.3%) of patients were ≤ 48 years of age, and 78 (75.7%) of patients were > 48 years of age. Out of 103 tumor samples, 16 (15.5%) were low grade (G1), 63 (61.2%) were intermediate grade (G2), and 24 (23.3%) were high grade (G3). Thirty (29.1 %) were T1 (T size ≤ 2 cm), 72 (69.9%) were T2 (T size 2.1 to ≤ 5 cm), and one (1%) were T3 (T size > 5.0 cm). The patient cohort comprised 85 (82.5%) N0 patients and 18 (17.5%) N1 patients. Eighty-four (81.6%) patients had invasive ductal carcinoma no special type (NST), seven (6.8%) had invasive lobular carcinoma, and 12 (11.6%) had other histological subtypes. Forty-nine (47.6%) had undergone breast-conserving surgeries like lumpectomy, WLE, etc. and 54 (52.4%) had undergone more extensive procedures like MRM, mastectomy, etc. The demographic and clinicopathological characteristics are summarized in Table 1.

**Table 1:** Patient and tumor characteristics G: grade; T: T size; LN: lymph node; NST: no special type; BCS: breast-conserving surgery

Clinicopathological Categories		Total Patient Number, n= 103	Percentage	
Age	≤ 48 years	25	24.3	
	>48 years	78	75.7	
Tumor grade	G1	16	15.5	
	G2	63	61.2	
	G3	24	23.3	
Tumor stage	T1	30	29.1	
	T2	72	69.9	
	T3	1	1	
Node status	N0	85	82.5	
	N1 (1 to 3 LN involvement)	18	17.5	
Histopathological diagnosis	Invasive ductal carcinoma, NST	84	81.6	
	Invasive lobular carcinoma	7	6.8	
	Other special histological types	12	11.6	
Surgery type	BCS	49	47.6	
	Mastectomy	54	52.4	
Median age	58 years			
Median tumor size	2.5 cm			

Comparison of CanAssist breast risk proportion between core needle biopsy (CNB) and surgical specimen (SS)

Using CNB as a sample, CAB stratified 24 (23.3 %) patients as high risk and 79 (76.7%) as low risk whereas 20 (19.4%) patients as high risk and 83 (80.6%) as low risk when SS was used as a sample. The proportion of CAB recurrence risk category distribution in various clinicopathological categories is summarized in Table 2.

**Table 2:** CAB risk category according to clinicopathological characteristics in CNB and SS (n=103) G: grade; T: T size; LN: lymph node; NST: no special type; CNB: core needle biopsy; SS: surgical specimen; CAB: CanAssist Breast test

Clinicopathological Category		Total	High Risk n, (%)	Low Risk n, (%)
Total cohort	CNB	103	24 (23.3%)	79 (76.7%)
	SS	103	20 (19.4%)	83 (80.6%)
Age	≤ 48 years - CNB	25	3 (12.0%)	22 (88%)
	≤ 48 years - SS	25	2 (8.0%)	23 (92%)
	> 48 years - CNB	78	21 (26.9%)	57 (73.1%)
	> 48 years - SS	78	18 (23.1%)	60 (76.9%)
Tumor grade	G1 - CNB	16	0 (0%)	16 (100%)
	G1 - SS	16	0 (0%)	16 (100%)
	G2 - CNB	63	9 (14.3%)	54 (85.7%)
	G2 - SS	63	5 (7.9%)	58 (92.1%)
	G3 - CNB	24	15 (62.5%)	9 (37.5%)
	G3 - SS	24	15 (62.5%)	9 (37.5%)
Tumor stage	T1 - CNB	30	4 (13.3%)	26 (86.7%)
	T1 - SS	30	2 (6.7%)	28 (93.3%)
	T2 - CNB	72	19 (26.4%)	53 (73.6%)
	T2 - SS	72	17 (23.6%)	55 (76.4%)
	T3 - CNB	1	1 (100%)	0(0%)
	T3 - SS	1	1 (100%)	0 (0%)
Node status	N0 - CNB	85	16 (18.8%)	69 (81.2%)
	N0 - SS	85	13 (15.3%)	72 (84.7%)
	N1 (1 to 3 LN involvement) - CNB	18	8 (44.4%)	10 (55.6%)
	N1 (1 to 3 LN involvement) - SS	18	7 (38.9%)	11 (61.1%)

Distribution of CanAssist Breast Risk Score in CNB and SS

**Figure 2:**
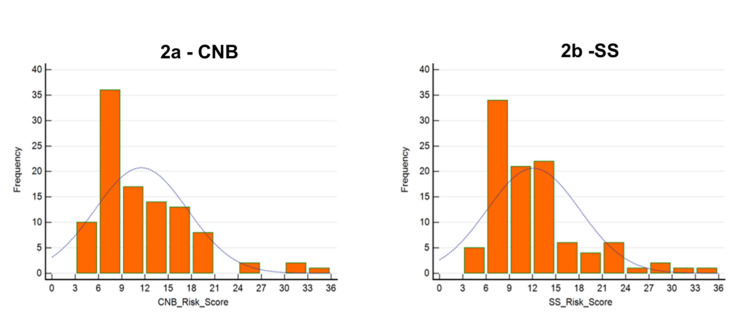
CAB recurrence risk score distribution in CNB versus SS in 103 cases. (2a) CNB versus (2b) SS. Figures were prepared using MedCalc Software version 22.032-64-bit. CND: core needle biopsy; SS: surgical specimen

Among the 103 patients, risk scores and risk categories were assessed for both CNB and SS. The mean CAB risk score for CNB was 11.5 (range 4.5 to 34.5) compared to 12.1 (range 4.4 to 33.4) for SS. These samples are representative of the spectrum of risk scores seen in CAB patient testing. The distribution of the CAB risk score is similar in both CNB and SS as is shown in Figure 2.

Concordance of CanAssist breast risk categories between CNB and SS

CAB was performed on 103 paired samples, with CNB being considered as a test assessment and SS as the gold standard. Low risk on stratification on CNB and SS was noted in 79 and 83 patients respectively and 77 patients were categorized as low risk on both CNB and SS (Table 3). On the other hand, 24 and 20 patients were stratified as high risk for recurrence respectively by using CNB and SS as a sample for CAB, and 18 patients were categorized as high risk on both CNB and SS (Table 3). The overall percentage agreement between the risk stratification category of CAB with CNB and SS was 92.2%. When SS was used as the gold standard, the concordance was 92.8% for the low-risk category and 90% for the high-risk category. Similarly, the percentage concordance for the low-risk category between CNB and SS was 97.5% and 75% for the high-risk category between CNB and SS, if CNB is considered the primary sample for the CAB test which is more likely to happen in the actual clinical practice.

**Figure 3:**
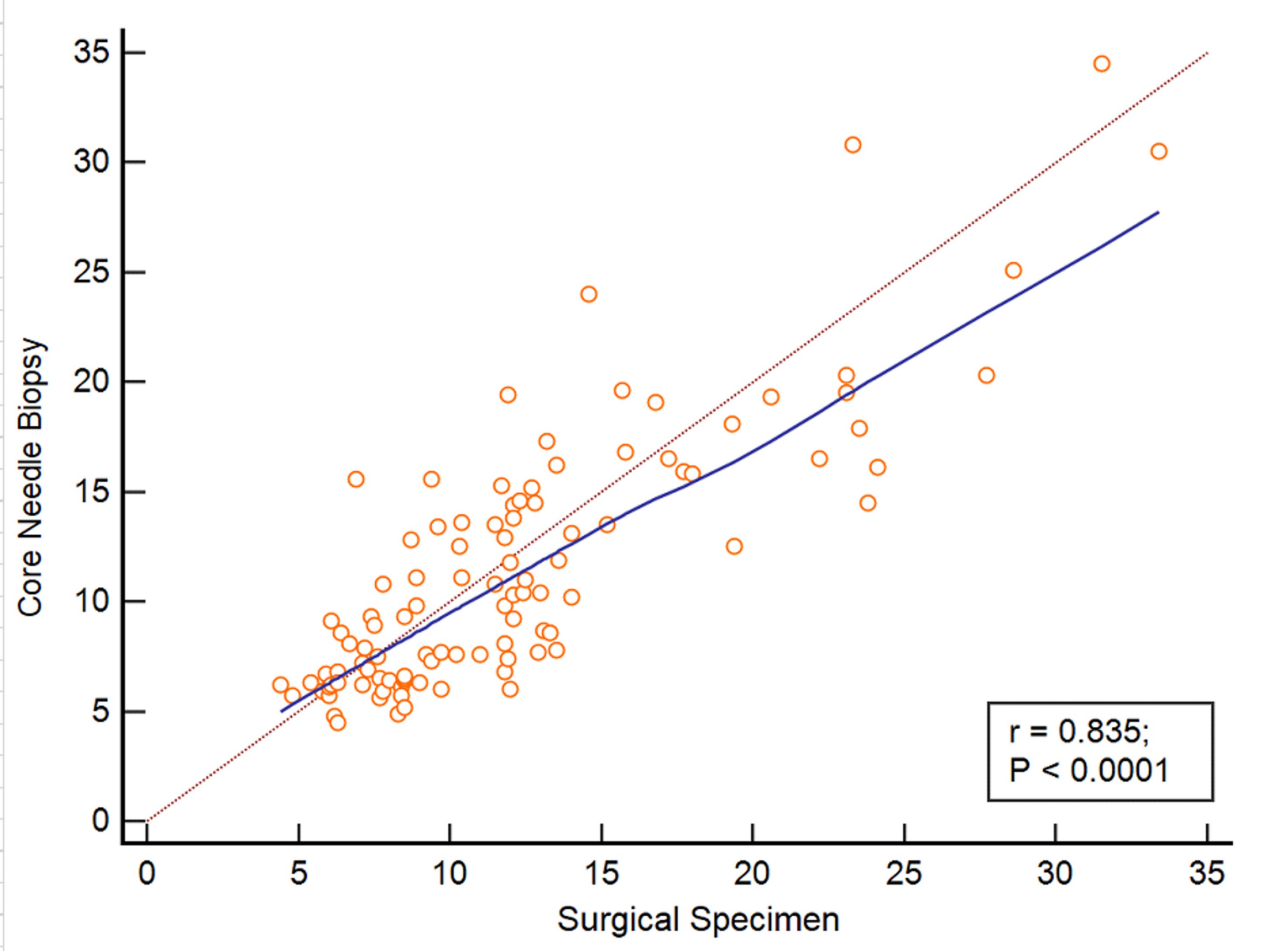
Scatter plot of CAB risk score on CNB versus SS. Correlation between CAB risk score from CNB versus matched SS sample. Pearson correlation coefficient 0.835 with a significance level of p < 0.0001. Figure prepared using MedCalc Software version 22.032-64-bit. CNB: core needle biopsy; SS: surgical specimen

**Figure 4:**
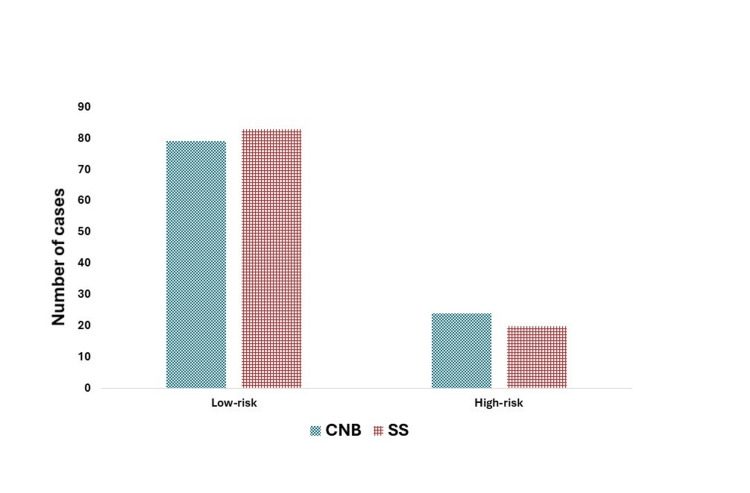
Risk category concordance between CNB versus SS Figure prepared using MedCalc Software version 22.032-64-bit. CNB: core needle biopsy; SS: surgical specimen

Upon further analysis, eight out of 103 cases showed discordant CAB risk categories between CNB and SS when SS was used as the gold standard. Of these six cases were stratified as high risk on CNB which were stratified as low risk on SS. Two cases were categorized as low risk on CNB, which were categorized as high risk on SS. The risk category concordance between CNB and SS is demonstrated in Table 3 and Figure 3. Pearson correlation analysis performed between the CNB and SS showed a strong correlation of 0.8351 (p< 0.0001) with a 95% interval for r (0.7653 - 0.8855) as shown in Figure 4.

**Table 3:** Comparison of CAB risk categories between CNB and SS CNB: core needle biopsy; SS: surgical specimen; CAB: CanAssist Breast test

SS CAB Risk Category	CNB CAB Risk Category				
		High Risk	Low Risk	Total	
	High risk	18	2	20	
	Low risk	6	77	83	
	Total	24	79	103	

Analysis of discordant cases

Detailed H&E review of the eight discordant cases in CNB and SS to assess the probable reasons for discordance with respect to the total number of cores in CNB, number of tumor-positive cores in CNB, and suboptimal fixation/processing in CNB and SS was performed. This review showed crush artifacts in two CNB samples and suboptimal fixation in two SS samples. In four samples no obvious cause was found and the likely reason could be under-sampling or tumor heterogeneity.

Accuracy of CAB with CNB

To evaluate the accuracy of CAB, sensitivity, specificity, NPV, and PPV were calculated using CNB as a test assessment and SS as the gold standard. Twenty cases categorized as high risk on SS were considered true positive and 83 cases categorized as low risk on SS were considered true negative. Six cases were stratified as high risk on CNB but low risk on SS were considered false positive. Two cases that were low risk on CNB but high risk on SS were considered false negative. For CNB, the sensitivity is 90.91%, specificity is 93.26%, PPV is 76.92%, NPV is 97.65%, and AUROC is 0.963. The data is summarized in Table 4.

**Table 4:** Accuracy of CAB with CNB PPV: positive predictive value; NPV: negative predictive value; AUROC: area under receiver operative characteristic

Variable	Percentage (%) in the Whole Cohort
Sensitivity	90.91
Specificity	93.26
PPV	76.92
NPV	97.65
Concordance	92.23
AUROC	0.963

Agreement with tumor volume

For all CNBs, tumor volume was calculated based on the number of tumor-positive cores multiplied by the average percentage of tumor content in all tumor-positive cores. Based on the tumor volumes derived, the CNB samples were bifurcated into two groups as follows: (1) Tumor volume < 2 units and (2) Tumor volume > 2 units. The inter-rater agreement (kappa) was studied for both groups. The inter-rater agreement (kappa) showed moderate agreement in the tumor volume group < 2.0 and improved to a strong agreement in the tumor volume group > 2.0. The findings are summarized in Table 5.

**Table 5:** Inter-rater agreement with tumor volume in CNB CI: confidence interval

Tumor Volume Group	Inter-Rater Agreement (Kappa)	95% CI
< 2.0	0.722	0.515 to 0.930
> 2.0	0.836	0.618 to 1.000

## Discussion

CNB has become the most frequently tested sample in BC. CNB is used for diagnosis, hormone receptor testing, and other biomarker tests that are being done to plan systemic therapy, including planning for NACT and NAHT. In many cases, CNB might be the only treatment naïve sample available for testing when a patient is treated with NACT/NAHT. SS has been used traditionally for histopathological tumor grading, staging, and predictive and prognostic factor testing. CAB has been validated analytically and clinically on FFPE tumor blocks from SS [7-12]. With the increasing use and importance of CNBs, there have been extensive publications describing studies analyzing the concordance of ER, PR, and Her2 between CNB and SS [17-19]. Concordance between CNB and SS is between 78.7 and 99.1% for ER, 73.5 and 95% for PR, and 56 and 98.8% for Her2 as described in the study by Rossi et al. [17]. Guidelines recommend clinical subtyping and hormone receptor testing (ER, PR, and Her2) on CNB despite concordance rates below 100% between paired CNB and SS, which is an acceptable practice worldwide [5]. Concordance studies are published for testing of prognostic and predictive multi-gene tests like Oncotype Dx, MammaPrint risk classification/Blueprint molecular subtyping, and EndoPredict which showcase that CNB provides an accurate and reliable evaluation of the molecular profile of invasive BC with results comparable to those from SS [20-22]. Qi et al. studied 50 matched paired samples and reported overall concordance in RS group classification between CNB and SS to be 74%, 72%, and 78% based on traditional cutoffs for Oncotype Dx [20]. Crozier et al. studied 121 paired CNB and SS samples and demonstrated 90.9% overall agreement for the Mammaprint Test [21]. Muller et al. have studied 40 paired samples and published Pearson r=0.92 with an overall agreement of 95% for EndoPredict [22]. These studies have demonstrated an increasing acceptance of CNB for biomarker, prognostic, and predictive testing. Also, there is an added benefit in the clinical management of these cases with respect to the choice of NACT/NAHT. These factors underlie the study of concordance in risk stratification by CAB when performed on CNB versus SS.

In this study of 103 paired samples of CNB and SS, an overall agreement of 92.2% was observed between the two types of samples, the concordance was 92.8% for the low-risk category and 90% for the high-risk category when SS was used as the gold standard. The ICC shows a strong correlation with r= 0.8351. In a real-world scenario, CNB is the first available sample and if tested the chances of SS being tested again are minuscule if any. Therefore, it is crucial to achieve high accuracy in the low-risk category, which would be treated only with endocrine therapy. In this study, discordant cases in which, there is a risk of under-treatment (low risk on CNB, high risk on SS) were only two, with concordance for the low-risk category between CNB and SS at 97.5%. This implies minimal adverse effect on decisions regarding the choice of adjuvant chemotherapy if CNB was used as the primary sample for the CAB test and is critical in the treatment of patients with early BC.

Risk proportions across various clinicopathological categories were similar as shown in Table 2. Whether CAB was performed on CNB or SS, in grade 1 and grade 3 cases the risk category concordance was 100%. Most discordances were observed in grade 2 tumors. Of the 63 grade 2 tumors, nine cases were stratified as high risk in CNB and five cases as high risk in SS. Fifty cases were categorized as low risk on CNB and 58 cases as low risk on SS. This is either because grade 2 is the major category (61.2%) or because these cases show more heterogeneity when compared to grade 1 or 3. Other clinicopathological categories like age (≤ 48 years or > 48 years) T size (T1 or T2) or node status (N0 or N1) showed acceptable high and low-risk proportions between CNB and SS. The similarity in the distribution patterns of CAB risk scores between CNB and SS as seen in Figure 2 also highlights the comparability of both sample types.

The H&E review of SS and CNB of discordant cases in our studies points to under-sampling, tumor heterogeneity, processing artifacts, and suboptimal fixation as the likely causes of CAB risk category discordance. Factors that play an important role in good concordance between CNB and SS are adequacy of sample representativeness. Adequate sampling, especially in larger tumors, is important, and retesting may be considered on SS in larger tumors. It has been suggested in studies that while taking cores for CNB, at least five samples should be taken including the center and periphery of the lesion to ensure adequate sampling of the tumor for heterogeneity [23]. If SS shows an increase in tumor grade or has evidence of morphological heterogeneity when compared to CNB, retesting of the CAB test on SS may be helpful. In our study, we noted that the presence of crush artifacts interferes with biomarker gradings, and it is advisable not to test CNB for samples showing crush artifacts. Heterogeneity, sampling artifacts, and under-sampling also allude to lower concordance in PR as described by Rossi et al. [17]. The effect of tumor volume on concordance was analyzed in our study and demonstrated, as expected, an improvement in concordance with increasing tumor volume in CNB (Table 5). Clark et al. have also reported increasing concordance with the increasing number of tissue cores in CNB [24].

Pre-analytical variables like cold ischemia time and total fixation time are essential factors for testing predictive factors. Guidelines have been issued for breast IHC tests for ER, PR & and Her2 testing with the recommendation of cold ischemia time of less than one hour and total fixation time of more than six hours and less than 72 hours in 10% neutral buffered formalin [25]. The pre-analytic fixation-related factors are most important to standardize interlaboratory results. As most centers find it easier to adhere to fixation guidelines for CNB, it has been observed that most CNB samples show better and prompter fixation with better retention of sample antigenicity. Few studies have concluded that CNB is more reliable for hormone receptor studies as prompt fixation helps avoid loss of antigens as in SS [26]. You et al. have concluded that CNB shows high diagnostic accuracy and good agreement for ER, PR, Her2, and Ki67 [19]. If the SS is found to have suboptimal tissue fixation (due to either unacceptable cold ischemic time or total fixation time), the addition of an optimally fixed CNB as a surrogate sample would help to reap the benefits of the CAB test.

This study is the first of its kind to compare the concordance of CAB risk stratification between CNB and SS. The high concordance in CAB risk categories between CNB and SS establishes CNB as a suitable alternative sample for the CAB test and would also be beneficial in patients where the SS shows suboptimal fixation/processing or when CNB is the only available sample. This study also paves the way for the use of the CAB test with CNBs for planning NACT and NAHT in patients with higher tumor burden.

The strengths of this study are the use of over 100 paired samples in a blind manner which allows us to determine concordance in an individual case reliably. Also, the samples tested cover the spectrum of early BC patients seen typically as well as risk scores seen in CAB testing. The limitations of this study are mainly the current lack of follow-up data on these patients to correlate the outcome with CAB risk stratification from CNB and SS samples. These patients are being followed up and will be reanalyzed at the end of 5 years with outcomes. We also have an ongoing prospective double-blind study about performing the CAB test on CNB for planning NACT or NAHT in pre and postmenopausal women. The results from both these studies would be conclusive in proving the utility of CAB in that setting.

## Conclusions

In summary, this study uses real-world data to evaluate the concordance of CAB risk stratification on paired tumor samples. The high concordance rates and good agreement of results between CNB and SS strongly support the utilization of CNB as a suitable alternative sample for the CAB test. Using optimally fixed and processed CNB samples with an adequate representative sampling of the tumor which would account for tumor heterogeneity would not only make the CAB test on CNB a reliable alternative to testing with SS but also help plan alternative neo-adjuvant therapies more effectively.
